# Embedded Ultrathin Cluster Electrodes for Long-Term Recordings in Deep Brain Centers

**DOI:** 10.1371/journal.pone.0155109

**Published:** 2016-05-09

**Authors:** Leila Etemadi, Mohsin Mohammed, Palmi Thor Thorbergsson, Joakim Ekstrand, Annika Friberg, Marcus Granmo, Lina M. E. Pettersson, Jens Schouenborg

**Affiliations:** Neuronano Research Center, Department of Experimental Medical Science, Lund University, Lund, Sweden; Monash University, AUSTRALIA

## Abstract

Neural interfaces which allow long-term recordings in deep brain structures in awake freely moving animals have the potential of becoming highly valuable tools in neuroscience. However, the recording quality usually deteriorates over time, probably at least partly due to tissue reactions caused by injuries during implantation, and subsequently micro-forces due to a lack of mechanical compliance between the tissue and neural interface. To address this challenge, we developed a gelatin embedded neural interface comprising highly flexible electrodes and evaluated its long term recording properties. Bundles of ultrathin parylene C coated platinum electrodes (N = 29) were embedded in a hard gelatin based matrix shaped like a needle, and coated with Kollicoat^™^ to retard dissolution of gelatin during the implantation. The implantation parameters were established in an *in vitro* model of the brain (0.5% agarose). Following a craniotomy in the anesthetized rat, the gelatin embedded electrodes were stereotactically inserted to a pre-target position, and after gelatin dissolution the electrodes were further advanced and spread out in the area of the subthalamic nucleus (STN). The performance of the implanted electrodes was evaluated under anesthesia, during 8 weeks. Apart from an increase in the median-noise level during the first 4 weeks, the electrode impedance and signal-to-noise ratio of single-units remained stable throughout the experiment. Histological postmortem analysis confirmed implantation in the area of STN in most animals. In conclusion, by combining novel biocompatible implantation techniques and ultra-flexible electrodes, long-term neuronal recordings from deep brain structures with no significant deterioration of electrode function were achieved.

## Introduction

Neural interfaces have the potential to provide key scientific tools to elucidate how the conscious brain functions at the cellular and network levels and to provide effective therapy for treatment of patients with neurodegenerative or psychiatric conditions [1–3]. However, the ability of current neural interfaces to record neuronal activity often deteriorates over time, limiting analysis of long term changes in neuronal activity [4, 5]. This instability depends at least partially on tissue movements relative to the electrodes, often termed micro-motions, and can occur if the active sites of the electrodes are unable to follow tissue movements caused by e.g. heartbeats, respiration or body movement [6–12]. Moreover, the electrode performance (i.e. signal-to-noise ratio) often deteriorates over time, presumably at least partly due to tissue reactions which functionally encapsulate the implant [13–17], as well as a loss of a substantial number of neurons adjacent to the electrode [11, 18–21]. Notably, the magnitude of glial scarring is dependent on micro-forces/micro-motions and the size of electrodes [22–27]. Thus, to mitigate these effects and thereby facilitate long-term recordings with sustained quality, it would likely be advantageous to use thin and flexible electrodes [26, 28–31].

To implant highly flexible electrodes deep into brain tissue, some form of structural support is necessary. While ultra-thin and therefore highly flexible electrodes can be implanted into deep tissue through a stiff cannula/guide tube, or by being glued onto a stiff guide [32, 33], these stiff supports need to be withdrawn after implantation to release the electrodes in the tissue and to let the tissue heal. Due to their size, such guides/cannulas cause additional stab wound-like injuries, and upon removal risk perturbing the position of the implanted electrodes. In addition, since tissue adheres to the guides/cannulas their withdrawal will create a drag force potentially disrupting the tissue [27].

The aim of the present study was to develop techniques to enable implantation of a bundle of ultra-flexible electrodes into deep brain targets without the need to use a guide or cannula for structural support, and to evaluate its long-term recording properties. To this end, bundles of ultrathin electrodes were embedded in a gelatin based matrix material shaped like a needle to allow easy penetration of the arachnoidea mater [30, 34]. To retard dissolution of the gelatin-based vehicle, the probe was coated with Kollicoat^™^. The coating thickness, as well as the timing of different steps during the implantation, were calibrated using an *in-vitro* model with mechanical properties similar to the brain [35–38]. Using this new technique we were able to implant and spread out ultra-thin and flexible platinum wires in the area of rat subthalamic nucleus (STN) and to evaluate the long-term (up to 8 weeks) functionality of the electrodes.

## Materials and Methods

### 1. Manufacturing and characterization of the electrode

#### 1.1 Probe fabrication and manufacturing

Thin, pure platinum (Pt) temper annealed microwires (Advent Research Material; England 12.5 μm) insulated with Parylene C (di-chloro-di-para-xylylene), were used as electrodes. Parylene C was chosen as the insulating material, since an intact, pore-free Parylene C coating is practically impermeable to water. In addition, Parylene C has previously been used as a surface coating material for implants in the medical device industry [39–42], it is highly biocompatible, anti-corrosive and extremely tolerant to the moist and ion-rich milieu encountered in the human body [39, 42] and has been shown to provide remarkable stability as a medical device-coating [42].

Parylenization was done using a Compact Bench Top Coating System (Labtop 3000, Para Tech Coating Inc., CA, US). Briefly, Pt-wires, were mounted on a custom-made metal frame and placed in a parylenization chamber in a Parylene Coating System (labtop 3000, Para Tech Coating Inc., CA, US). The Parylene coating procedure was done in three steps: vaporization, pyrolysis and deposition. Parylene C was vaporized in vacuum at 165°C. At the initial stage Parylene C is in the dimeric form (di-para-xylylene). Vaporization was followed by pyrolysis at 650°C to produce reactive monomers (para-xylylene). In the final deposition step the monomeric gas was cooled down to 220°C, where it polymerizes into poly-para-xylylene and forms a uniform insulating coat on the Pt-wires.

**Fig 1:**
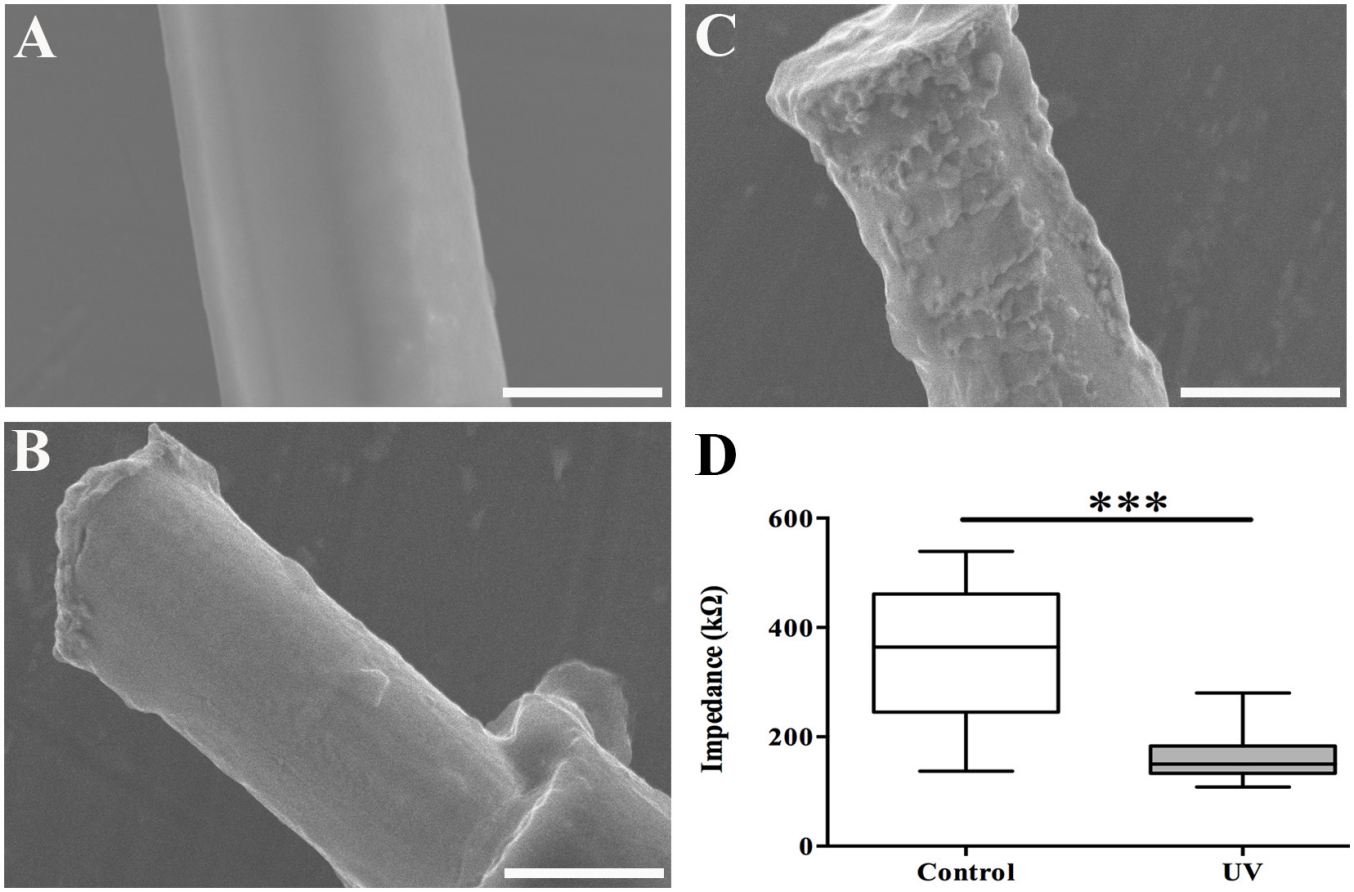
Visualization of the electrode-surface. (A) SEM image of parylene coated Pt wire. (B) De-insulation with low power UV resulted in a smooth surface of the electrode. (C) Irradiation with high power UV resulted in an increment in the geographical surface area without increasing the physical area. (D) The electrode impedance as measured at 1 kHz was reduced after irradiation with high power UV (***p < 0.001). (Scale bar A = 11μm, B, C = 10 μm).

In this study, a 4 μm thick coat of Parylene C was polymerized on the surface of the Pt-wires. A scanning electron microscope (SEM) (SU1510-ver1.0 model, Hitachi High-technology corporation, Japan), was used to evaluate the properties and thickness of the parylene coating. The SEM-assessment of the quality of the Parylene C coating showed a smooth surface, free of cracks or holes, and with a thickness of 4±1μm (Fig 1A). The impedance of the insulation was tested by measuring the impedance of a 4 cm long Pt-wire loop, insulated with 4 μm Parylene C using Gamry Potentiostat (Series G300, Warminster, USA). The un-insulated tips of the loop were connected to a working electrode and impedance was measured against a large surface platinum counter electrode and Ag/AgCl reference electrode in 0.9% saline solution. The value for the impedance exceeded 21 MOhm at 1 kHz frequency indicating an intact Parylene coating.

Twenty-nine Pt-wires, insulated with Parylene C, were soldered to a printed circuit board (PCB) containing 30 gold-plated pads. To obtain a high quality soldering, a thin layer of gold was electroplated on the de-insulated proximal ends of the Pt-wires before they were soldered to the PCB. One of the 29 wires was reserved as a local reference. The remaining 28 wires were used as recording electrodes. A non-insulated silver wire (diameter: 150 μm, Advent research materials Ltd, England) was soldered to the PCB and used as animal ground during recordings.

The electroplating setup consisted of a cathode (the Pt-wires connected to a PCB) and a gold anode dipped in a gold plating potassium cyanide bath (1g Au/100ml; Sargenta AB, Malmö Sweden). The set-up was immersed in a sonicator. Voltage—controlled electroplating at 0.3V for 10 min with continuous sonication at 38 kHz was performed using an ultrasonic transducer (Emmi-20HC, EMAG technologies, Germany). After completion of the electroplating procedure, further sonication was performed for an additional 10 minutes, to remove any loosely attached nanoparticles [43].

Parylene C coated wires were de-insulated and cut at the tips using high-precision laser (Standard micro-milling system, New Wave Research Class 1, USA). In short, the wires were collected, using polyethylene glycol (PEG 1000) dissolved in ethanol (30%W/W), placed between two glass cover slips for immobilization, and the peripheral ends were spread out on a flat surface. The following parameters were used for de-insulation: 90% low UV energy (wavelength 355nm and energy density 1.89 J/cm^2^), 50 Hz pulse frequency, target square 60μm/ x 20 μm, 1 second pre-warming, 2 passes with 20 μm/s scanning speed of the target square; and for cutting: 90% high UV (wavelength 355 nm and energy density 6.3 J/cm^2^), 50 Hz frequency, 50 μm x 20 μm target square. To reduce impedance, the surface area of the de-insulated tips was increased by briefly boiling the tips using a focused laser 85% high UV (wavelength 355 nm and energy density 5.95 J/cm^2^) 60 μm x 20 μm target square with, a 50Hz pulse frequency and 100 μm/s scan speed (Fig 1C). A Gamry Potentiostat was used for in vitro measurement of the interface impedance at a frequency of 1 kHz. Individual conductive leads were measured with respect to a large surface platinum counter electrode and an Ag/AgCl reference electrode in 0.9% NaCl solution at room temperature.

#### 1.2 Moulding and embedding of the probe

A plexiglas mould was manufactured to enable controlled embedment of the ultrathin wires and shape them into a straight and stiff matrix-embedded probe with a sharp tip. To this end, a custom made replica of the final desired probe shape was made in brass. The replica was subsequently pressed between two pieces of heated plexiglas (210°C) to make an imprint in the plexiglas and thus shape a mould. Precise and tight fit of the two halves of the mould was accomplished by closing it using 6 fitted screws. The gelatin matrix solution was prepared by dissolving 3g of gelatin B (VWR international, Sweden) in 7 mL de-ionized water at 70°C [34]. PEG 400 (750 mg) (Sigma-Aldrich Chemie GmbH, Germany) and glycerol 150 mg (VWR, BDH Prolabo, France) were added to the gelatin solution as filling and plasticizing agent respectively. The final concentration of the gelatin solution was 1.4% glycerol, 6.9% PEG and 27.5% gelatin. The electrode bundle was slowly dipped into the warm gelatin solution (50°C), at a controlled speed of 1.2 cm/min, using a custom-made voltage controlled motorized micro-manipulator. After drying for 1–2 min, the electrode bundle was centered in the mould and an additional volume of heated gelatin solution was injected through the side channel in the mold. The mould containing the probe was kept in a humidor chamber with the relative humidity (21%) optimized for slow (48h) drying of the gelatin matrix. The dried electrode bundle was mechanically cleaned from excessive gelatin. To increase dissolution time of the gelatin based matrix after implantation, two layers of Kollicoat^™^ MAE 100P (Supplier: Sigma Aldrich Sweden AB (5% in absolute ethanol) were added, to avoid dissolution of the gelatin, by dip coating at a speed of 6 cm/min using the same custom-made motorized micro-manipulator as mentioned above. Kollicoat^™^ MAE 100P is a dispersible polymer, derived from methyl acrylic acid/ethyl acrylate, often employed as a film-forming agent by pharmaceutical companies

#### 1.3 In-vitro tests of dissolution time and electrode spread

The effect of Kollicoat^™^ coating on dissolution time of the gelatin was evaluated in-vitro. Dummy probes of gelatin, i.e. moulded gelatin needles without any internal electrodes, were made by injecting a gelatin solution (1.4% glycerol, 6.9% PEG and 27.5% gelatin, cresyl violet 5% W/V dissolved in MilliQ water) into the mould used for probe-fabrication. Cresyl violet was added to the gelatin matrix to increase the visibility of the probes during the implantation-procedure and thereby facilitate the visual observation of the probe insertion track and gelatin dissolution. The moulds containing the dummy gelatin probes were dried in a humidor chamber with the relative humidity 21% for 48h, as described above.

**Evaluation of dissolution time:** The dummy probes were divided into two groups i.e., kollicoated (5%, 2 layers, n = 8) and non-kollicoated controls (n = 7). The dissolution of dummy gelatin probes was evaluated during insertion in 37°C, 0.5% agarose which is commonly used as an *in vitro* model of mechanical properties of the rat brain [38]. The dummy probes were implanted to depth of 7 mm into the agarose with a speed of 100 μm/sec using a micromanipulator (Kopf, Model 2650, California, US). The whole implantation procedure and 10 min post implantation was documented using a microscope (Kaps, Asslar/Wetzlar, Germany), with a connected camera filming the procedure (Infinity 2-1RC model, Lumenera, made in Canada). The onset of gelatin dissolution followed by deviation from the intended straight track line was visually estimated by two observers by replaying the film second by second.

**Evaluation of electrode spread:** In the subsequent *in-vitro* experiment, we used two layers of 5% Kollicoat^™^ and examined its effect on the spreading of the electrode wires when inserting the electrode bundle to the full target-depth of 8mm, corresponding to the approximate depth of the STN in rats. In this experiment, the electrodes were implanted into the 37°C, 0.5% agarose in a three-step procedure. First, the probe was inserted 7 mm into the agarose with a speed of 100μm/sec, using a micromanipulator. Second, we paused for 10 min, allowing the gelatin-embedding to dissolve. Finally, the probe was advanced 1 mm further at the speed of 10 μm/sec, allowing the distal tips of the wires to spread and form a cluster of recording-sites at the target-depth of 8mm. The final spread was assessed from pictures documented with the camera connected to the microscope. The final spread was defined as the largest distance between the distal parts of the wires.

#### 1.4 Mechanical testing of microwires

The buckling test (Zwick GmbH & Co. KG, Materials Testing Machine with load cell Zwick/Roell KAP-Z (0, 04-4N), Acquisition system Zwick/Roell testXpert II) was performed on 10 mm long wires (n = 10) with fixed endpoints. The maximum average force for deformation (Fmax, at which the wire begins to bend) was below the detection limit (0.001 N). The theoretical buckling force was therefore calculated using Euler’s formula, given by:

$$F=\frac{\pi^{2}EI}{{\left(KL\right)}^{2}}$$ (1)

where E is the elastic modulus of Pa (platinum’s E = 168 GPa), I is area moment of inertia, L is the effective length of the wire (10 mm) and K is the effective length factor of the wire (0.5 when both ends are fixed). The theoretical value of maximum deformation force was found to be 79.5 μN. The low value of theoretical buckling force for the microwires used indicates high flexibility.

### 2. *In-vivo* studies

#### 2.1 Experimental animals

Female Sprague-Dawley rats weighing 200-250g, (Taconic, Denmark) were used. All rats received food and water ad libitum and were kept in a 12-hour day—night cycle at a constant environmental temperature of 21°C and 65% humidity. All procedures were approved in advance by the Malmö/Lund Animal Ethics Committee on Animal Experiments (registration number M95-11), regulated by the code of regulations of the Swedish Board of Agriculture. These regulations, including directives from the European Union, follow the law on animal welfare legislated by the Swedish parliament. The animals were kept in the animal facilities of the Biomedical Center at Lund University and experiments were carried out at the Section for Neurophysiology.

#### 2.2 Implantation of electrodes

The rats were anaesthetized with intraperitoneal (i.p.) injections of fentanyl (0.3 mg/kg; Braun, Germany) and medetomidine hydrochloride (Domitor Vet, 0.3 mg/kg; Orion pharma, Finland), the head was shaved and animals were mounted in a stereotactic frame for electrode implantation in the area of the STN. During the entire time of surgery, the eyes of the animals were kept moist by opthalmic ointment of Sodium hyaluronate 2.0mg/ml (ZilkEye, Bohus Bio Tech AB, Strömstad, Sweden). Implantation was made at the following co-ordinates in relation to bregma; AP: -3.6mm, L = ± 2.4mm, at a depth of 7.8 mm. Briefly, the skin in the surgical area was disinfected with 70% ethanol and an incision was made along the midline, the skin was retracted and the periosteum removed. Burr holes were drilled though the cranium. The dura was removed after land marking of the dorso-ventral co-ordinates (see above). The electrode bundle was implanted into the brain using the same three-step procedure as described in the *in-vitro* tests (Section 1.3).i In short, the probe was advanced to the pre-target depth (1 mm above the STN) at a speed of 100 μm/s using a micromanipulator. Following a pause of 10 minutes, the probe was slowly advanced an additional 1mm into the STN at a speed of 10 μm/s (i.e. the last 1mm was traversed in 1 min and 36 sec). The ground wire was wound around stainless steel skull screws (anchor screws 1x2 mm, Agnthos Inc Lidingö, Sweden) positioned in burr holes in the skull bone, so as to get in contact with CSF. The electrode PCB was attached to the skull using dental cement (GC FujiCEM2, GC Belgium, Europe), anchored to the stainless steel screws attached to the skull bone. After surgery, the rats received subcutaneous injections of Temgesic (buprenorphine, 50mg/kg body weight) to reduce postoperative pain, and antidote to the anesthesia (Antisedan, atipamezole hydrochloride, 0.5 mg/kg body weight). Animals were monitored during the awakening phase.

#### 2.3. Neural recordings and data analysis

Neural recordings were performed in anaesthetized rats (1% Isoflurane, Isoba^®^vet., Abbott Laboratories Ltd, Berkshire, England; n = 12). The recordings commenced 1-day post implantation. The rats were connected to a Plexon data acquisition system (Plexon Inc, Texas, USA) via a head stage and pre-amplifier for in-vivo recordings. Each session of extracellular recordings lasted approximately 10 min, and recordings were made approximately once a week for up to 8 weeks post-implantation. Signals were band-pass filtered between 250 Hz to 8000 Hz. One animal was excluded as the electrode crown fell off during handling of the animal. Three animals were excluded from further electrophysiological analysis since the histological analysis showed that they did not hit the target STN.

*The noise level* for a given recording was estimated using the median absolute deviation (MAD) estimator for the standard deviation, given by:

$$\sigma_{N}=median\left(\frac{\left|v\left(n\right)\right|}{0.6745}\right)$$ (2)

where |v(n)| is the absolute value of the band-pass filtered digital signal [44]. Recordings in which the estimated noise level was within 0.3 to 2 times the mean noise level across all recordings were included in the analysis. For recordings that did not meet these criteria, the corresponding electrodes were deemed as not working [45]

*Spike detection* was carried out by applying a negative threshold corresponding to four times the estimated standard deviation (Eq 2) and spike waveforms were temporally aligned on the point of maximum amplitude of the detected peak [46].

*Spike sorting* was performed using the first six principal component analysis (PCA) weights as features [47]. The number of units present in a given recording was estimated by fitting the PCA weight distribution to Gaussian mixture models with one to six mixture-components (clusters/units) and selecting the model at which the Bayesian information criterion (BIC) converged to a minimum value [48]. The selected model was then used to cluster the data, and spikes whose maximum posterior probability of belonging to any of the cluster was less than 0.8 were labeled as outliers [45]. All sorting results were verified visually in PCA space and the time-domain to correct occasional mistakes in finding the number of clusters. The signal-to-noise ratio (SNR) of putative single-units was calculated as:

$$SNR=\frac{s_{pp}}{2\;\cdot \sigma_{R}}$$ (3)

where s_pp_ is the peak-to-peak amplitude of the unit’s mean waveform and *σ*_*R*_ is the standard deviation of the residuals after subtracting the mean waveform from each detected spike waveform.

Putative single-units were finally validated based on their mean-waveforms (polarity, shape, signal-to-noise ratio and amplitude) and firing statistics (percentage of inter-spike intervals shorter than 1 ms). In short, thresholds for the waveform-related features were set empirically to reject putative units with waveforms that were not considered sound from a physiological perspective. Also, putative units with an ISI-violation rate above 0.5% were rejected.

Apart from the visual inspection of spike-sorting results and the visual supervision of the unit validation procedure, the entire processing chain was carried out automatically. This automatization was implemented in order to ensure as much consistency as possible in the data-processing and thereby allow the direct comparison of spike sorting results for recording sessions at different points in time [49].

#### 2.4. *In-vivo* Impedance measurements

*In-vivo* impedance at 1kHz was measured once a week during the eight-week period in four of the animals. A Plexon stimulator 2.0 (Plexon Inc, Texas, USA) was used to generate a ± 100 nA 1kHz sinusoidal current, and impedance was measured by monitoring the changes in voltage across the electrode. The electrode impedance is influenced by the properties of the electrode-tissue interface, increasing with a progressive immune reaction [50]. Thus, stability of impedance over time indicates a stable electrode-tissue interface and/or a moderate immune reaction.

#### 2.5. Lesioning and tissue preparation

Before sacrificing the animals, electrolytic lesion marks were made for a subset of the electrode tips (6 wires/electrode bundle) to provide an indication of the spread of the wires inside the brain tissue. Lesions were induced sequentially through separate wires (1 mA, 2 ms) in deeply anesthetized rats, as per [51], using a DC-stimulator (Digitimer, Class I, model DS3, made in the UK). Rats were subsequently euthanized with an i.p. overdose of sodium pentobarbital (200mg/kg; Apoteket Product and Laboratories Incoch Laboratorier AB, Stockholm Sweden) and transcardially perfused with 150 ml of ice cold 0.9% saline followed by 300 ml of ice cold 4% paraformaldehyde (PFA) in 0.1M phosphate buffer, pH 7.4. The brains were dissected and post-fixed overnight in 4% PFA at 4°C, after which they were repeatedly rinsed and cryoprotected in 20% sucrose and finally frozen.

### 3. Histology

#### 3.1. Staining of tissue

To verify the location of the electrode wires in relation to the target area in the brain, as well as estimating the in vivo spread of the wire bundles, histological examination of the tissue was performed. The frozen brains were sectioned coronally (30 μm thickness) using a cryostat. Sections were mounted on Super Frost^®^ plus slides (Mänzel-Gläser, Germany) and stained with cresyl violet. In short, sections were immersed in ethanol/chloroform (1:1) overnight at room temperature. The following day slides were rehydrated in decreasing concentrations of ethanol (100%, 95%), rinsed in distilled water (2 minutes/step), stained with cresyl violet (Life Science products & services company; 0.1% in 0.3% acetic acid in dH_2_O; 5 minutes) and rinsed in distilled water (1 minute), followed by dehydration in ethanol (95%, and 2 x 100%), and xylene (2 x 100%, 5 minutes). Finally, slides were cover slipped-using DPX mounting media (Fluka, Germany). Slides were examined and light microscopy images were captured using a DS-Ri1 digital camera (Nikon Instruments, Japan) mounted on a Nikon eclipse 80i microscope.

## Results

### 1. Electrodes used

As verified using SEM, the de-insulation resulted in a clean metal surface and we chose to de-insulate the distal section extending 25 μm (±5 μm) from the tip (Fig 1B and 1C). Focused high intensity UV irradiation of the distal de-insulated electrode-tips caused transient boiling of the platinum and resulted in an increase in surface area and a significant reduction in electrode impedance at 1kHz (mean reduction 55%, p < 0.001 Mann-Whitney non-parametric test) compared to the preboiled controls (Fig 1C and 1D).

### 2. Implantation vehicle—in vitro studies

An important aim of the present work was to develop an implantable gelatin embedded bundle of ultra-flexible electrodes that can be made to spread out in deep targets in the brain. To avoid using very rapid insertion velocities, which can cause damage to the tissue, we needed to significantly increase the time for dissolution of the gelatin vehicle. To solve this problem the embedded microelectrodes were coated with a double layer of Kollicoat^™^ (a single layer of Kollicoat was insufficient, data not shown). The presence of the Kollicoat^™^ coating significantly increased the onset of dissolution-time in vitro at 37°C from mean and standard deviation (SD) of 37±29 sec for the non coated gelatin probes, to 114±75 sec for the kollicoated probes (p < 0.05, Student t-test).

**Fig 2:**
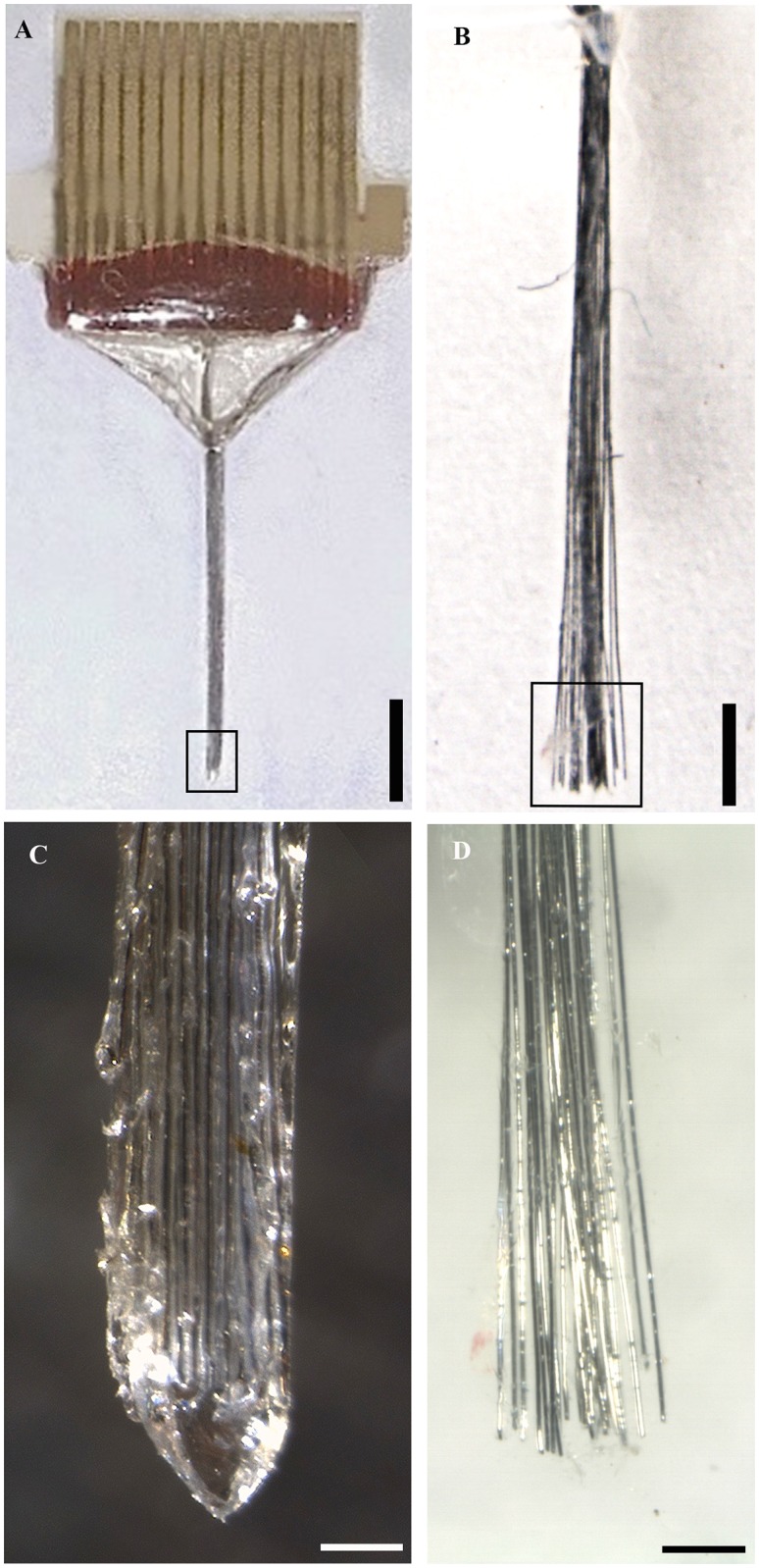
Light field images visualizing the cluster electrode before and after in vitro implantation. (A) The kollicoated gelatin embedded electrode bundle (diameter 250 μm) before implantation in agarose, (B) Electrode after implantation in agarose visualizing the straight implantation track, (C) Close up of the distal part of the probe and its conical tip (D) Close up of the electrode tip visualizing the spread of the electrode bundle wires after insertion into 37°C agarose. The final spread was approximately 500 μm (Scale bar A = 1mm, B = 2 mm, C = 150μm, and D = 250 μm).

The increase in dissolution-time rendered by the kollicoating was sufficient to maintain mechanical support of the gelatin embedded, needle shaped flexible wire-bundle (Fig 2A and 2C) during the implantation procedure to the pre-target depth of 7 mm at a speed of 100μm/sec. Following a pause of 10 min, to allow dissolution of the gelatin based material, the microelectrodes were further advanced 1 mm at speed of 10 μm/sec and successfully spread out (Fig 2B and 2D). The mean and SD spread in agarose was 515 ± 69 μm.

### 3. Electrode performance in vivo and histology

The kollicoated gelatin probe (diameter 250 μm) used in vivo was shaped as a needle with a conical tip to reduce dimpling of the cortical surface. The same implantation method as developed in vitro was used. Eight animals, for which the implanted probe could be confirmed to reach the area of STN (histology, see below), were used for electrophysiological analysis. The overall median percentage of working channels (according to noise level) was 96.36% (IQR = 12.95%). The percentage of working channels appeared to be stable over time.

#### 3.1 Noise level, impedance and signal to noise ratio

**Fig 3:**
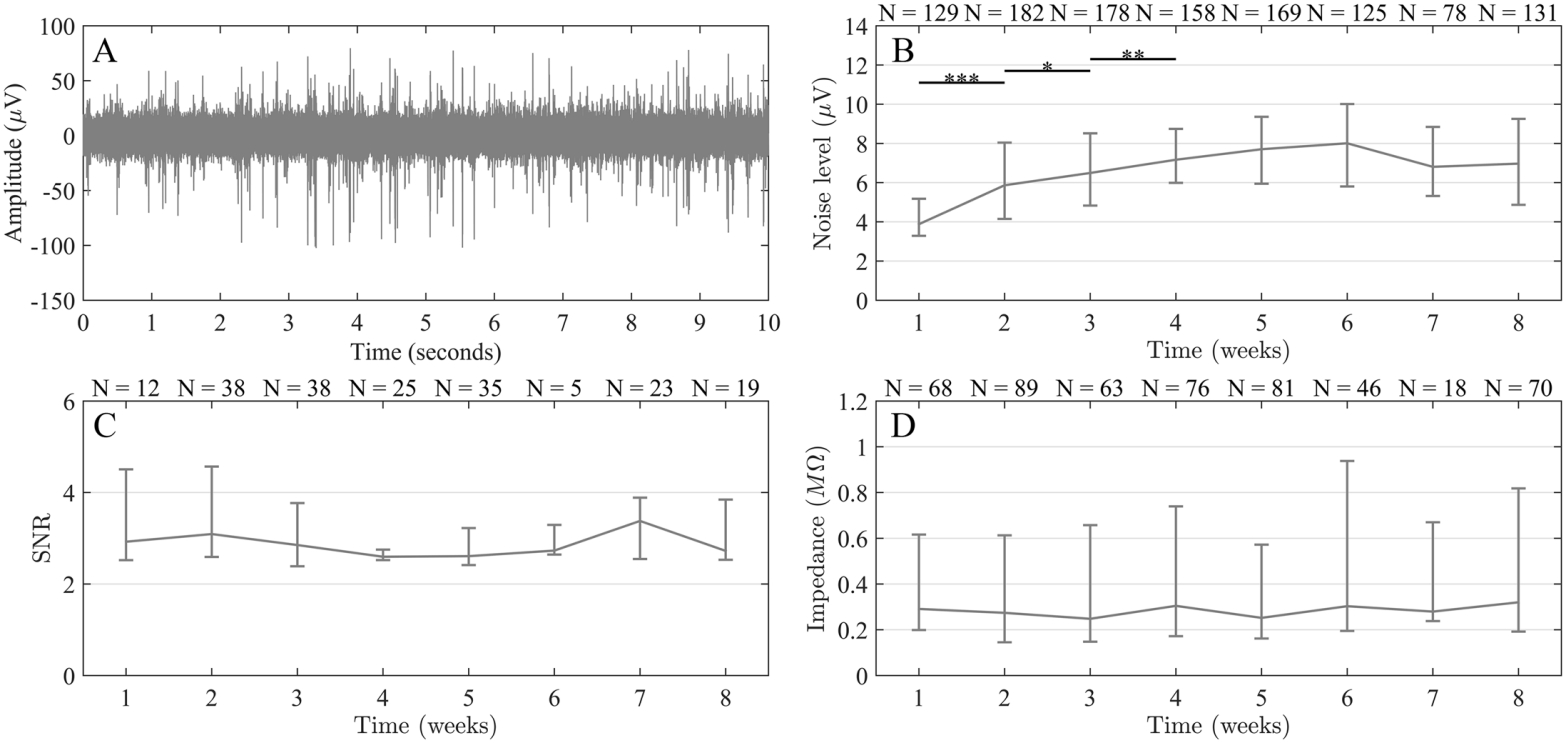
In-vivo characterization of electrode performance. (A) A 10 seconds long segment of an example recording made 8 weeks after implantation, illustrating the general characteristics of the recordings. (B) Distribution (median and interquartile range, IQR) of noise level across all recordings made within each week. Weekly noise level increased between weeks 1 and 2 (***p < 0.001), 2 and 3 (*p < 0.05) and 3 and 4 (**p < 0.01). After that, the weekly noise level remained stable (p > 0.05). N refers to the number of working channels recorded from each week. (C) Distribution (median and IQR) of SNR of recorded channels with identified single-units. SNR remained stable (p > 0.05) throughout the experiment. N refers to the number of working channels with identified single units within each week. (D) Distribution (median and IQR) of in-vivo impedance of recorded and working channels. Impedance remained stable (p > 0.05) throughout the experiment. N refers to the working channels on which an impedance measurement was performed each week. The variation in N (B-D) is mostly due to a varying number of animals recorded from each week.

Over the entire course of the experiment (up to 8 weeks; recordings starting 1–2 days after implantation), the overall median noise level of working channels was 6.63μV (IQR = 3.80 μV, IQR = Interquartile range). Fig 3A shows a 10 sec long segment of an example recording (high pass filtered) to illustrate the normal appearance of recordings. The recording was performed during week 8 after implantation. Fig 3B shows the distribution of noise level across all working channels in all animals recorded from each week. The weekly median noise level increased from week 1 to 2 (p < 0.001, Mann-Whitney U test), week 2 to 3 (p < 0.05), and from week 3 to 4 (p < 0.01). After this initial period, noise levels did not change from week to week. Fig 3C shows the distribution of SNR of the highest-SNR unit across all channels recorded from each week, only considering channels on which units were identified. At 51% of the implanted electrode sites, single-units were identified at some point in time during the course of the experiment. The weekly median SNR did not change significantly (p > 0.05) between consecutive weeks during the full duration of the experiment. The overall median SNR was 2.77 (IQR = 1.50). Fig 3D shows the distribution of *in-vivo* impedance across all channels in four of the animals (see Section 1.4) as a function of time after implantation. Throughout the experiment, no significant changes in weekly median *in-vivo* impedance were observed (p > 0.05) indicating a relatively stable electrode-tissue interface. The overall median impedance was 276 kΩ (IQR = 522 kΩ).

#### 3.2 Histological assessment of track line and spread of wires in vivo

**Fig 4:**
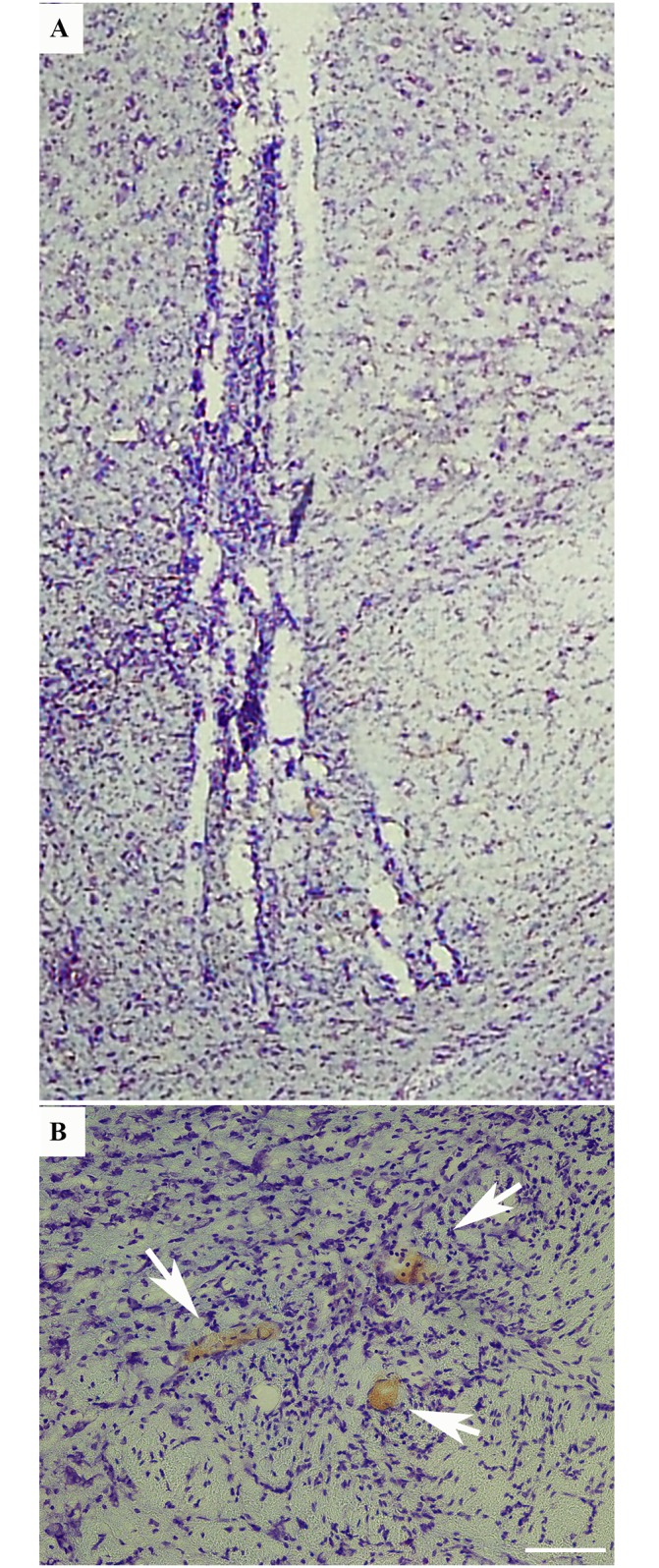
Light field micrographs of track line and spreading of electrodes *in vivo*. (A) Electrode tip spread in with an average spread of approximately ≈ 500 μm in lateral direction. (B) Micrographs of electrically induced lesion in STN indicating the positions of separate electrode tips. Lesion marks induced by electrical stimulation of separate wires (6 wires/electrode, AC current, 1mA, 2 ms indicate the positions of the separate electrode tips in brain sections stained with cresyl violet.

We could confirm successful targeting of the STN in 8 of the 11 animals. It should be noted that, because of the spread, some of the wires were positioned a bit anterior or posterior to the STN. Implantation of electrodes was achieved through a narrow track line, with the spread of the distal wires in anterio-posterior and lateral axes (Fig 4A). Electrically induced lesions were used to further indicate the positions of separate electrode tips, allowing us to verify the position within the STN. The mean and SD of the *in vivo* spread was 497 ± 146 μm which is about the width of the rat STN (Fig 4A and 4B).

## Discussion

The development of this new neural interface was prompted by the need to obtain durable multichannel recordings from deeply located brain structures. As a step towards this goal, we developed and characterized a novel multichannel neural probe composed of ultrathin and individually independent wires, embedded in a gelatin matrix, forming a needle-like probe. To implant this probe, we developed a three-step procedure resulting in the introduction of a cluster of ultrathin and flexible electrodes in the intended deep target tissue. Importantly, apart from an increase in noise over the first 4 weeks, we found no obvious deterioration of electrode properties, i.e. in vivo impedance and SNR were relatively stable, during the 8-week post-implantation period.

### On the implantation method developed

In this study we, for the first time, accomplished implantation of ultrathin electrodes into deep brain targets without using rigid guides or cannulas. By assembling the electrodes into a bundle and support them in hard gelatin, a temporary rigidity sufficient for implantation into superficial structures such as the cortex cerebri, has previously been achieved [30]. However, one problem with gelatin is that it quickly gets hydrated during implantation and thus loses its rigidity. To be able to reach deep targets without using a very high insertion speed, a factor known to cause injuries of for example blood vessels [19, 52, 53], we here developed a coating-technique, using Kollicoat^™^ to transiently retard water penetration and thus the dissolution of the gelatin matrix. To calibrate the probe properties, initial *in vitro* experiments were made in tempered agarose (37°C, 0.5%) which is known to have similar mechanical properties as brain tissue [35]. To account for variability in dissolution time, we used a Kollicoat^™^-concentration which would be able to seal the gelatin from moisture and therefore hold together the electrodes during a time sufficient to advance the electrode bundle to a depth corresponding to the intended pre-target depth. We then paused for a sufficient amount of time (10 minutes) to ensure dissolution of the gelatin, and thus allow the electrode wires to spread during further advancement (1mm) of the bundle into the agarose model. Subsequent *in vivo* experiments confirmed the feasibility of the method. However, it should be noted that this implantation technique is critically dependent on many parameters, including the speed and depth of insertion, temperature, and shape and dimensions of the probe. Hence, a recalibration of the coating thickness may be necessary in case the electrodes are to be implanted e.g. at another depth than in the present study.

By using a highly precise mould, which ensures that the probe is centered and well aligned from tip to proximal end, a straight insertion track was possible to obtain. To target the STN area in the *in vivo* experiments we used the coordinates described in [54] and calculated a track-line from the position of the Bregma on the skull. While this method provides a first approach in order to assess the accuracy of the method, it is by no means an exact method for implantation and will consequently result in variations in the final position reached due to anatomical variations between animals. In the present study, 8 of 11 implants did hit the target, the other implants were either found above or below the target. To more precisely establish the target coordinates before implantation, imaging of the brain using e.g. MRI, is useful. When implanting electrodes in the clinic, this problem is routinely mitigated by imaging the target brain nuclei and adjusting the coordinates with respect to the reference system of a stereotactical frame [55].

### On the long term performance of the ultrathin electrodes in vivo

A common problem with available electrodes is that the recording performance usually deteriorates over time [12, 56]. It is commonly assumed that this deterioration is caused by the tissue response towards the implant, resulting in glial scarring and loss of neurons nearby [16, 57, 58]. The present findings of stable SNR and impedance over an 8 week period add support to the notion that durable long term recordings of good quality can indeed be obtained provided that ultrathin and ultra-flexible electrodes are used [59]. In addition, recent findings indicate that gelatin embedding can significantly reduce the microglia reactions and also counteract the loss of neurons near the implant [26, 30]. Hence, the use of a truly biocompatible material as an implantation vehicle may have contributed significantly to the stability in impedance and signal quality seen in the present study. Another feature, which may influence long-term stability, is the surface structure of the de-insulated part of the electrode tips. It is commonly assumed that the SNR of neural recordings depends upon the impedance of the electrode which is related to e.g. the tissue response and the electrode surface area [60–63]. Many different methods have therefore been developed to reduce the impedance of the electrodes, including electro-plating and the use of charge carrying polymers [43, 64]. However, such coatings are not always physically stable [65, 66] resulting in alterations of impedance not related to for example degree of glial scarring. In the present study, we developed a simple and robust method, employing high-power UV laser, to enlarge the surface area of the recording sites without significantly increasing their geometrical surface area. It is conceivable, but remains to be tested, if such surface enlarged electrodes are able to improve long-term signal quality.

### Clinical applications

Apart from research purposes, durable long-term recordings are also likely to be of interest for closed loop deep brain stimulation (DBS) [67] and for detecting the onset of abnormal activity such as epileptic attacks [68–70]. In conventional DBS, a constant stimulation regime is applied after an initial evaluation of stimulation parameters. Enabling a more dynamically adjustable stimulation, which takes into account the local effects of the stimulation and also the current brain state, has the potential to improve the DBS technology significantly [71–73]. A requirement for reliable closed loop DBS is that stable recordings can be obtained for long periods of time. Although not evaluated in this study, the current probe may also be adapted to be used for stimulation of deep brain targets. In such cases it may be advantageous to further increase the size of the de-insulated distal part of the micro-wires to increase the charge delivery capacity.

### Limitations and future directions

The present design is aimed at an initial in vivo evaluation of the long-term performance of ultrathin electrodes implanted in a deep brain target. While the results regarding stability of impedance, noise and signal quality are promising, it is clear that there is room for improvement of the probe design e.g. with respect to the number of electrodes and the embedding material. For example, while we chose 29 electrodes in the current design of the cluster electrodes, for practical purposes the number of individual electrodes may have to be increased considerably. In some cases, an even greater spread of electrodes will be beneficial. As for the embedding medium, the possibility to incorporate anti-inflammatory drugs which further protect the target tissue [74] may be useful.

### Conclusions

The novel design presented here combines ultra-flexible electrodes with a biocompatible implantation vehicle, thus enabling introduction of a cluster of multiple ultrathin and ultra-flexible electrodes into a deep brain target tissue. The finding that the recording quality did not deteriorate significantly during 8 weeks indicates that it is indeed possible to achieve stable recordings in deep brain structures for long periods of time.

## Supporting Information

S1 File Data for statistical analysis. The supporting information contains data used for statistical analysis and are arranged in a corresponding way to the presentation of data in the result section, i.e., *In vitro* impedance, Dissolution time, Electrode statistics, Noise level, Impedance, and SNR. In addition, there is a section (Sheet name: “Key”) for interpreting the data arrangement. The file is organized using Microsoft excel.
(XLSX)
